# Immune checkpoints in thymic epithelial tumors: challenges and opportunities

**DOI:** 10.1016/j.iotech.2019.09.002

**Published:** 2019-09-16

**Authors:** Nicolas Girard

**Affiliations:** Head of the Thoracic Oncology Program, Institut du Thorax Curie-Montsouris, Institut Curie, Paris, France

**Keywords:** Thymoma, Thymic carcinoma, Chemotherapy, Immunotherapy, Autoimmune disorder, Myasthenia

## Abstract

Thymic malignancies are rare mediastinal cancers, classified according to the World Health Organization's histopathologic classification which distinguishes thymomas from thymic carcinomas. One key consideration when discussing immunotherapy for thymic epithelial tumors is that one-third of patients diagnosed with thymomas present at the time of diagnosis with autoimmune disorders, the most common being myasthenia gravis. The first step in the understanding of autoimmunity in thymic epithelial tumors is to distinguish true autoimmune disorders from paraneoplastic syndromes; besides pathophysiology, clinical correlates, impact on oncological management and survival may differ strongly. Autoimmune disorders are related to a deregulation in the physiological role of the thymus (i.e. to induce central tolerance to tissue self-antigens) through control of differentiation and subsequent positive and negative selection of immature T cells; from a clinical standpoint, in thymomas, once autoimmune disorders are present, they may not regress significantly after thymectomy. PD-L1 expression, while observed in more than 90% of epithelial cells of the normal thymus with a medullar tropism respecting Hassall's corpuscles, has also been identified in thymomas and thymic carcinomas using various immunohistochemistry protocols. Immune checkpoint inhibitors of the PD-1/PD-L1 axis have been assessed in advanced and metastatic thymic epithelial tumors, mainly thymic carcinomas. Several case reports have been published, and four trials have assessed the efficacy and safety of these inhibitors. Immunotherapy is not standard given the frequent occurrence of severe autoimmune disorders, and clinical trials are ongoing.

## Highlights

• Thymic malignancies are rare tumors.
• Advanced, metastatic and refractory thymic tumors may be treated with chemotherapy.
• Autoimmune disorders, such as myasthenia gravis, are observed in 30% of cases.
• PD-L1 expression is a hallmark of thymic epithelial cells.
• Immunotherapy is not standard, given the occurrence of autoimmune disorders.

## Thymic epithelial tumors: current treatment strategies

Thymic malignancies are rare mediastinal cancers, classified according to the World Health Organization's histopathologic classification which distinguishes thymomas from thymic carcinomas [1]; approximately 1500 patients are diagnosed every year in Europe [2]. Thymomas reproduce the architecture of the normal thymus, combining epithelial tumor cells with non-tumoral lymphocytes, and are further subdivided into subtypes (A, AB, B1, B2 and B3) based upon the degree of cell atypia, the relative proportion of the lymphocytic component and the resemblance to normal thymic architecture [1]. Thymic carcinomas are similar to their extrathymic counterparts, with the most common subtype being squamous cell carcinomas that harbor specific immunohistochemical (expression of CD5 and CD117) and molecular features. These differ from thymomas and squamous cell carcinomas originating from the lung or other organs [3,4]. However, neuroendocrine carcinomas may occur in the thymus as primary tumors. The staging of thymic tumors is currently based on the 8th edition of the American Joint Committee on Cancer/Union for International Cancer Control TNM staging classification which integrates macroscopic findings but is only assessed after surgical resection, as the classification of early-stage tumors requires information about pathological invasion of the capsule and the perithymic fat [5].

The management of thymic epithelial tumors requires cooperation between clinicians, surgeons and pathologists from establishing the diagnosis to organizing the multimodal therapeutic strategy [6]. Surgery is the mainstay of curative-intent treatment in limited-stage tumors, as complete resection represents the most favorable prognostic factor for overall survival in both thymomas and thymic carcinomas [7]. Postoperative radiotherapy is debated after complete resection of thymomas, but has been associated with better outcome in thymic carcinomas [8]. Systemic treatment may be delivered in a curative-intent approach for patients presenting with locally advanced tumors at the time of diagnosis, with invasion of intrathoracic neighboring structures and/or dissemination to the pleura and the pericardium, precluding complete resection. In such cases, chemotherapy aims to reduce the tumor burden – possibly allowing subsequent surgery and/or radiotherapy – to achieve prolonged disease control [10,11].

For the treatment of unresectable, metastatic and recurrent tumors, which are more frequently thymic carcinomas than thymomas, systemic agents are used in a more palliative-intent setting. Historically, several consecutive lines of chemotherapy may be administered when the patient presents with tumor progression [9,10]. Recent real-life evidence provides landmark efficacy data for such strategies [10]. Targeted agents are included in the strategy, mainly consisting of the antiangiogenic agent sunitinib [11] and the MTOR inhibitor everolimus [12]; exceptional cases of thymic carcinoma harbor a druggable oncogenic alteration, mainly described in the *KIT* and *PI3KCA* genes [[13], [14], [15]], with opportunities for the use of specific inhibitors in a precision medicine strategy.

This review focuses on the key immunological features of thymic epithelial tumors that are relevant in the clinic for the treatment of patients with regards to the frequent occurrence of autoimmune disorders in conjunction with thymomas, and recent results with immune checkpoint inhibitors based on potential biomarkers.

## Autoimmune disorders in thymic epithelial tumors

One key consideration when discussing immunotherapy for thymic epithelial tumors is that one-third of patients diagnosed with thymomas present at the time of diagnosis with autoimmune disorders, the most common being myasthenia gravis [6,[16], [17], [18]]. Other frequent disorders include pure red cell aplasia (5% of cases) and hypogammaglobulinemia (5% of cases) (Table 1).

**Table 1:** Autoimmune disorders associated with thymomas

Neuromuscular	Myasthenia gravis
	Myotonic dystrophy
	Limbic encephalitis
	Peripheral neuropathy Autonomic neuropathy
	Acquired neuromyotonia Morvan syndrome (neuromyotonia and encephalitis)
	Stiff person syndrome Cerebelar degeneration Polymyositis (carcinomas)
Hematologic disorders	Red cell aplasia
	Pernicious anemia
	Erythrocytosis
	Pancytopoenia
	Hemolytic anemia
	Leukemia
	Multiple myeloma
Collagen and autoimmune disorders	Systemic lupus erythematosus
	Rheumatoid arthritis
	Sjogren syndrome
	Scleroderma
	Interstitial pneumonitis
Immune deficiency disorders	Hypogammaglobulinemia (Good syndrome)
	T-cell-deficiency syndrome
Endocrine disorders	Multiple endocrine neoplasia
	Cushing syndrome
	Thyroiditis
Dermatologic disorders	Pemphigus
	Lichen planus
	Chronic mucosal candidiasis
	Alopecia areata
Miscellaneous	Giant cell myocarditis
	Nephrotic syndrome
	Ulcerative colitis
	Hypertrophic osteoarthropathy

Bernard C, Frih H, Pasquet F, et al. Thymoma associated with autoimmune diseases: 85 cases and literature review. Autoimmun Rev 2016; 15:82–92.

Bouchet ME, Dansin E, Kerjouan M, et al. B004. OS01.04. Prevalence of autoimmune diseases in thymic epithelial tumors insights from RYTHMIC. Mediastinum 2017:4 doi: 10.21037/med.2017.AB004

Padda SK, Yao X, Antonicelli A, et al. Paraneoplastic syndromes and thymic malignancies: an examination of the International Thymic Malignancy Interest Group Retrospective Database. J Thorac Oncol 2018; 13:436–46.

From a practical clinical standpoint, one recommendation of the Clinical Practice Guidelines of the European Society for Medical Oncology when thymic epithelial tumor is suspected is to conduct a systematic immunological check-up (including complete blood cell count with reticulocytes and serum protein electrophoresis, and anti-acetylcholine receptor and antinuclear antibody tests), record a complete history and conduct a full clinical examination (looking particularly at neurological signs) [6]. This is to diagnose the most common autoimmune disorders that may impact any therapeutic intervention, including surgery, radiotherapy and chemotherapy, and potential eligibility for immunotherapy.

Autoimmune disorders are usually observed in thymic carcinomas, and also in combined tumors with a thymoma component. However, pure carcinomas may be associated with true paraneoplastic syndromes related to the direct secretion of cytokines or hormones by tumor cells.

The largest report of the features of paraneoplastic and autoimmune disorders comes from the retrospective cohort of 6670 patients registered in the International Thymic Malignancy Interest Group database [18]. The results of this analysis confirm the previously reported significant association between autoimmune disorders and lymphocyte-rich, type B1 thymomas, early stage of the disease, and complete resection of the tumor; autoimmune disorders are also associated with a tendency towards a better outcome in terms of recurrence and survival.

One issue with all cohort studies is that checks for less common autoimmune disorders may not have been conducted systematically for all patients, especially in the absence of symptoms. Metachronous autoimmune disorders may also be underestimated, given the surgical orientation of the majority of databases with potentially limited follow-up of patients. Meanwhile, differential diagnosis may be challenging with postoperative treatment complications, as chemotherapy may produce cytopenias, and peripheral neurologic or cardiac side-effects. The absence of significant prognostic value of autoimmune and paraneoplastic disorders may suggest association with more aggressive tumors, as suggested by a higher rate of chromosomal alteration reported recently using data from the Cancer Genome Atlas [15]. This may be overcome by favorable clinical factors including: (i) earlier diagnosis of the tumor in the case of baseline autoimmune and paraneoplastic disorders with a higher chance of complete resection; (ii) more aggressive locoregional treatment, including thymectomy, which is known to be effective in myasthenia gravis regardless of the presence of a thymic tumor [19]; and (iii) closer clinical follow-up with potentially earlier diagnosis of recurrences.

## Pathogenesis of autoimmunity in thymic epithelial tumors

The first step in the understanding of autoimmunity in thymic epithelial tumors is to distinguish true autoimmune disorders from paraneoplastic syndromes; besides pathophysiology, clinical correlates, impact on oncological management and survival may differ strongly.

**Figure 1:**
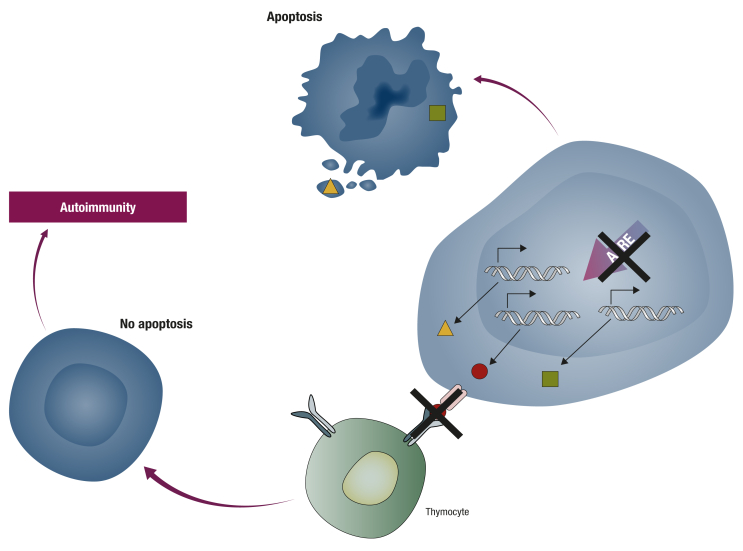
Inhibition of autoimmune regulator leads to release of autoreactive lymphocytes and autoimmune disorders. Adapted from Mathis D, Benoist C. Aire. Annu Rev Immunol 2009; 27:287–312.
• thymic carcinogenesis may be associated with genetic changes that impair the development of T cells, and generate an increased number of self-reactive lymphocytes [24].

Autoimmune disorders are related to a deregulation in the physiological role of the thymus (i.e. to induce central tolerance to tissue self-antigens) through control of differentiation and subsequent positive and negative selection of immature T cells. This phenomenon, while primarily active during childhood, still exists in adults [20]. This process is deregulated along with thymic carcinogenesis, as:

• immature thymoma-derived lymphocytes may escape the disorganized tumor micro-environment, disrupting the journey through the thymic medulla where self-tolerance is primarily induced [21,22];
• medullary thymic epithelial cells have defects in antigen presentation related to loss of expression of the transcription factor AIRE (autoimmune regulator), similar to that described in autoimmune polyendocrinopathy candidiasis ectodermal dystrophy. AIRE has the unique capability to express all self-tissue-related antigens at the cell surface of thymic epithelial cells of the medulla, and the inactivation of AIRE leads to the absence of expression of some antigens and the release of self-reactive lymphocytes outside the thymus (Figure 1) [23]; and

These mechanisms are not exclusive, as highlighted by the fact that AIRE is not inactivated in type B1 thymomas which are more frequently associated with autoimmune disorders [25], and patients can develop an autoimmune disease after thymectomy. The Cancer Genome Atlas analysis reported the overexpression of acetylcholine receptor and other related genes in myasthenia-positive thymomas, suggesting that defective negative T-cell selection is unlikely to be the sole autoimmunizing mechanism [15].

The role of PD-1/PD-L1 interaction in the non-neoplastic thymus is not fully understood; this interaction negatively regulates beta selection and modulates the positive selection [24]. PD-1 is involved in CD8+ T-cell tolerance through peripheral intrinsic mechanisms such as deletion or functional inactivation, and facilitates the peripheral differentiation of CD4+ T cells into regulatory T cells (Treg). Activation of lymphocytes through metabolic pathways is observed in expression profiling of thymomas [4].

From a clinical standpoint, in thymomas, once autoimmune disorders are present, they may not regress significantly after thymectomy.

Paraneoplastic disorders generally result from production of hormones, cytokines or peptides by tumor cells that lead to metabolic derangements; other mechanisms may include cross-reaction between tumor neoantigens and tissue-related antigens, with peripheral induction of autoantibody production stimulated by the presence of the tumor [26]. Thus, successful treatment of the underlying tumor often improves paraneoplastic syndromes, such as in neurological disorders like Lambert-Eaton myasthenia syndrome which is caused by autoantibodies to voltage-gated calcium channels in the presynaptic neuronal cell membrane, and often observed in thymic carcinomas [27]. Differential diagnosis between Lambert-Eaton syndrome and myasthenia gravis may be challenging for thoracic oncologists, especially if autoantibodies are not tested systematically. This may explain the number of patients with thymic carcinomas reported as having ‘myasthenia’.

## Immune-related biomarkers in the thymus and thymic tumors

PD-L1 expression, while observed in more than 90% of epithelial cells of the normal thymus with a medullar tropism respecting Hassall's corpuscles [28], has also been identified in thymomas and thymic carcinomas using various immunohistochemistry protocols (Table 2) [[29], [30], [31], [32], [33], [34], [35], [36], [37], [38], [39], [40], [41], [42], [43], [44], [45], [46], [47]]; overall, expression of PD-L1 is common in thymomas and thymic carcinomas, and is usually high and intense. In the micro-environment, a large series of 100 thymomas and 69 thymic carcinomas [35] reported high expression of PD-L1, IDO and FOXP3 Tregs in 36%, 13% and 16% of cases of thymoma, respectively. High expression of PD-L1, IDO and FOXP3 Tregs was associated with a higher grade of tumor histology. In patients with thymic carcinomas, high expression of PD-L1, IDO and FOXP3 Tregs was identified in 36%, 14% and 29% of cases, respectively.

**Table 2:** Selected studies assessing PD-L1 expression by tumor cells in thymomas and thymic carcinoma

	Technique, antibody	Thymomas		Thymic carcinoma	
		(*n*)	PD-L1 positive (*n*, %)	(*n*)	PD-L1 positive (*n*, %)
Katsuya et al. [29]	TMA, clone E1L3 (H-score, 1% of tumor cells cut off)	101	22 (23%)	38	26 (70%)
Padda et al. [30]	TMA, clone 5H1 (intensity high)	65	44 (68%)	4	3 (75%)
Arbour et al. [31]	Slides, clone E1L3 (25% of tumor cells cut off)	12	11 (94%)	11	4 (34%)
Yokohama et al. [32,33]	Slides, EPR1161 (H-score, 20% of tumor cells cut off)	82	44 (54%)	25	20 (80%)
Weissferdt [34]	Slides, clone E1L3 (5% of tumor cells cut off)	74	47 (64%)	26	14 (54%)
Markevski et al. [28]	Slides, clone SP142 (1% of tumor cells cut off)	38	35 (92%)	8	4 (50%)
Wei et al. [35]	TMA, clone E1L3 (% of cells and intensity)	100	100 (100%: 36% low, 64 high)	69	69 (100%: 64% low, 36% high)
Guleria et al. [36]	TMA, clone SP263 (1–25% of tumor cells cut off)	84	69 (82%)		
Suster *et al.* [37]	TMA, clone SP142 (1–50% of tumour cells cut off)			21 (lymphoepithelioma like histology)	15 (71%: 67% high, 33% low)
Tiseo et al. [38]	TMA, clone E1L3 (H-score, 1% of tumor cells cut off)	87	16 (20%)	25	13 (52%)
Bagir et al. [39]	Slides, clone AM26531AF-N (intensity)	37	21 (57%)	6	4 (67%)
Sakane et al. [40]	Slides, clones SP142, SP263, 22C3, Dako 28-8 (50% of tumor cells cut off)			53	26–49 (49–92%)
Hakiri et al. [41]	Slides, clone SP142 (50% of tumor cells cut off)	81	22 (27%)		
Chen et al. [42]	TMA, clone SP142 (% of cells and intensity)	50	24 (48%)	20	14 (70%)
Terra et al. [43]	Slides, clone SP263 (1–50% of tumor cells cut off)	11	10 (91%)	6	2 (33%)
Owen et al. [44]	Slides, clone 22C3 (% of tumor cells)	32	26 (81%)	3	3 (100%)
Bedekovics et al. [45]	slides, clone SP142 (1–50% of tumor cells cut off)	29	20 (69%: 70% high, 30% low)	7	6 (86%: 17% high, 83% low)
Duan et al. [46]	Slides, clone Ab58810 (% of cells and intensity)	13	13 (100%: 46% high, 64% low)	20	20 (100%: 65% high, 35% low)
Funaki et al. [47]	Slides, multiple clones (% of cells)			43	26 (60%)

TMA, tissue micro-array.

The significance of this finding as a signal of active immune response against thymic epithelial tumor cells and thus a rationale for the assessment of immune checkpoint inhibitors targeting PD-1 or PD-L1 remains debatable, given: (i) the high frequency of PD-1 and PD-L1 expression in the non-neoplastic thymus; (ii) the fact that all data were actually obtained in surgically resected early-stage tumors, and not from specimens of metastatic or advanced tumors that are those potentially eligible to immune checkpoint inhibitors; (iii) in thymomas, the presence of immature and mature T cells surrounding tumor cells is part of the prototypic architecture, and not a marker of actual antitumor response; and (iv) the potential immune modulation induced by chemotherapy, radiotherapy or targeted agents, such as reported with sunitinib, that may lead to occurrence or worsening of autoimmune disorders [11], which is part of the standard treatment strategy in advanced disease, and may lead to modulation of PD-L1 expression in immune cell populations including Tregs.

In a series of 43 patients with thymic carcinomas, PD-L1 increased in patients who received induction chemotherapy, and the change was strongly correlated with epithelial mesenchyma transition status [47]. In another series, the expression of PD-L1 in tumor cells differed between primary and metastatic or recurrent tumors and between metastatic/recurrent tumors at different time points in almost 20% of cases [43].

**Figure 2:**
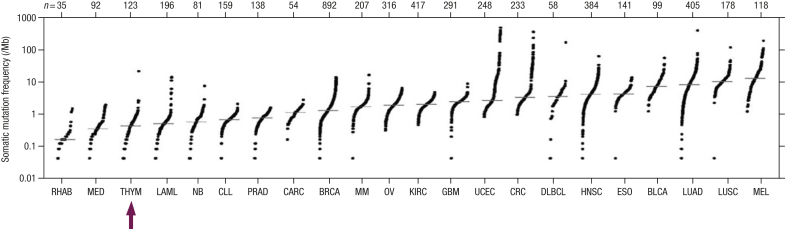
Tumor mutation burden in thymic epithelial tumors. Reprinted from Radovich M, Pickering CR, Felau I, et al. The integrated genomic landscape of thymic epithelial tumors. Cancer Cell 2018; 33:244–258; RHAB, Rhabdoid tumors; MED, Unveal melanoma; THYM, Thymic malignancies; LAML, acute myeloid leukemia; NB, Neuroblastoma; CLL, chronic lymphoid leukemia; PRAD, Prostate adenocarcinoma; CARC, adrenocortical carcinoma; BRCA, breast invasive carcinoma; MM, Malignant Mesothelioma; OV, Ovarian carcinoma; KIRC, kidney renal papillary cell carcinoma; GBM, Glioblastoma Multiform; UCEC, uterine corpus endometrial carcinoma; CRC, Colorectal cancer; DLBCL, Diffuse Large B Cell Lymphoma; HNSC, Head and Neck Squamous Cell Carcinoma; ESO, esophageal carcinoma; BLCA, bladder urothelial carcinoma; LUAD, Lung Adenocarcinoma; LUSC, Lung Squamous Cell Carcinoma; MEL, melanoma.

The tumor mutation burden is emerging as a potential biomarker for immunotherapy in solid epithelial tumors. Data from the Cancer Genome Atlas and Foundation Medicine indicated a low tumor mutation burden in thymomas and thymic carcinomas; only 6% of carcinoma cases had >10 mutations/Mb and 3% had >20 mutations/Mb (Figure 2) [13,15].

## Immunotherapy in thymic malignancies

Approximately 20–30% of thymomas and 70–80% of thymic carcinomas present with unresectable, recurrent and/or metastatic disease [6]. Current options include cytotoxic combination regimens, combining platin with anthracyclins, etoposide or taxanes, and targeted agents such as sunitinib; response and survival rates are usually limited, ranging from 20% to 30% and from 6 to 7 months, respectively [10]. Based on results obtained in other solid, refractory tumors in adults, especially of squamous cell differentiation, and possibly considering PD-L1 expression observed in thymic epithelial tumor cells – despite its intrinsic and constitutive relationship with the thymic origin of cancer cells – immune checkpoint inhibitors of the PD-1/PD-L1 axis have been assessed in advanced and metastatic thymic epithelial tumors, mainly thymic carcinomas. Several case reports have been published, and four trials have assessed the efficacy and safety of PD-1/PD-L1 inhibitors in patients with advanced thymic epithelial tumors (Table 3) [[48], [49], [50], [51], [52], [53], [54], [55], [56], [57], [58]].

**Table 3:** Reported results of anti-PD-1/PD-L1 in thymic malignancies

	Thymomas				Thymic carcinoma				Grade ≥3 adverse events	
	*n*	Response	Stable disease	Outcome	*n*	Response	Stable disease	Outcome	Rate	Events
		*n* (%)	*n* (%)			*n* (%)	*n* (%)		*n* (%)	
**Single case reports**										
Zander et al. [48]	1	1 (100%)	0 (0%)	Response at 2 months					1 (100%)	Cutaneous toxicity involving skin, mouth, esophagus, uvea and glans
Isshiki et al. [49]					1	1 (100%)		Response at 9 weeks	0 (0%)	
Uchida et al. [50]					4	3 (75%)	1 (25%)	Response at 10, 12 and 16 weeks	1 (25%)	General malaise
**Clinical trials**										
Giaccone et al. [51]					40	9 (23%)	21 (53%)	mPFS: 4.2 months mOS: 24.9 months	6 (15%)	Myositis, myocarditis, pancreatitis, hepatitis and pemphigoid
Cho et al. [52]	7	2 (29%)	5 (72%)	mPFS: 6.1 months	26	6 (23%)	13 (50%)	mPFS: 6.1 months	9 (27%)	Myositis, myocarditis, myasthenia and hepatitis
Katzuya et al. [53]					15	0 (0%)	11 (73%)	mPFS: 3.8 months	2 (13%)	Elevated transaminases and adrenal insufficiency
Rajan et al. [54]	7	4 (57%)	2 (28%)	NR	1	0 (0%)	1 (100%)	NR	5 (71%)	Myositis

mPFS, median progression-free survival; mOS, median overall survival; NR, not reached.

### Clinical trials

The landmark evidence comes from a phase II trial with pembrolizumab, a fully humanized IgG4 antibody that targets the PD-1 receptor, in 40 patients with thymic carcinomas. Forty-eight percent of cases had squamous cell differentiation, and 15% of cases had a neuroendocrine phenotype [28]. The tumors were metastatic in 33 patients (median of two metastatic sites), the median number of previous systemic therapies was two, and 52% and 58% of patients had previously been operated on or had received chest radiotherapy, respectively: this suggests that the patients had been heavily treated, had a relatively limited burden of disease and had a good performance status of -0 or 1 in 95% of cases. Any history of autoimmune disease or other malignancy requiring treatment led to exclusion from this study. Pembrolizumab 200 mg was given every 3 weeks. Of the 40 patients, six (15%) developed serious autoimmune disorders: two cases of polymyositis and myocarditis (complete recovery with steroids but requirement of a pacemaker for complete auriculo-ventricular block – this correlated with the presence of T-cell-receptor clones in muscle that increased in blood with treatment with pembrolizumab); one case of pancreatitis, hepatitis and type 1 diabetes mellitus; one case of bullous pemphigoid, (recovered with steroids); one case of polymyositis and hepatitis; and one case of transaminitis. Three patients had to discontinue treatment after an adverse event. In this trial, the response rate was 23%: there was one complete response, eight partial responses and 21 (53%) patients with stable disease. The median duration of response was 23 months. Median progression-free and overall survival were 4.2 and 24.9 months, respectively. PD-L1 expression – using immunohistochemistry with DAKO 22C3 antibody – was observed in ≥50% of tumor cells for 10 patients, six of whom had a response to pembrolizumab; only three of the 27 patients with PD-L1 expression by tumor cells <50% had a response. Interferon gamma signature assessed using the Nanostring assay correlated with response to pembrolizumab.

Further analysis was conducted to assess the tumor mutation burden assessed by targeted sequencing as a predictor of response; this was not found to be predictive [55]. Single alterations are being investigated further.

A second trial was conducted in Korea with a similar design [52]. Of 33 patients enrolled, 26 had thymic carcinomas and seven had thymomas (four type B1, one type B2/B3 and one type B3). Three patients had a history of myasthenia gravis (considered to be controlled), 30% of patients were treated beyond third-line treatment, and 36% of patients had a history of mediastinal radiotherapy. With regards to efficacy, two (29%) patients with thymomas had a partial response, and five (72%) patients had stable disease. Of 26 patients with thymic carcinomas, five (19%) had a partial response and 14 (54%) had stable disease. The median progression-free survival was 6.1 months for both groups. Five (71%) of seven patients with thymomas and four (15%) of 26 patients with thymic carcinomas reported grade ≥3 immune-related adverse events, including hepatitis, myocarditis, myasthenia gravis (some patients had pre-existing myasthenia gravis), thyroiditis, antineutrophil cytoplasmic antibody-associated rapidly progressive glomerulonephritis, colitis and subacute myoclonus; treatment of these side-effects was mainly based on steroids and immunoglobulins. Only high PD-L1 expression was predictive of a response. Subsequent analyses showed that the proliferative response of peripheral blood PD-1+CD8+ T cells, measured as the fold change in the percentage of Ki-67+ cells 7 days after treatment, may be a useful surrogate biomarker for predicting response and prognosis [].

A phase II trial with nivolumab, an IgG4 antibody that targets the PD-1 receptor, was conducted in Japan for patients with thymic carcinomas [53]; 15 patients were accrued in the first stage, which aimed to identify at least one patient with a response. No response was actually observed. However, 11 patients had stable disease, including five patients for ≥24 weeks. Median progression-free survival was 3.8 months. As the early termination criterion was fulfilled during the first stage, patient accrual was terminated. As in other trials, patients were heavily pretreated. Only two patients presented with severe autoimmune disorders (elevated transaminases and adrenal insufficiency); it remains unclear whether these data, together with the pembrolizumab data, suggest correlation between efficacy and the occurrence of side-effects.

The fourth trial was a phase I trial with avelumab [54,56], a fully human, IgG1 anti-PD-L1 antibody. This trial reported data on eight patients: seven with thymomas (two type B3, one type B2/B3, two type B2 and one type B1) and one with thymic carcinoma. Two patients with thymomas had a confirmed partial response, two had unconfirmed responses, two (including the patient with thymic carcinoma) had stable disease, and one had progressive disease. Interestingly, three patients responded after a single dose of avelumab. Treatment-related adverse events were immune-related events, including myositis, in five patients, precluding continuation of avelumab. The trial was discontinuated. Correlative studies suggested that avelumab induces infiltration of the tumor by macrophages, natural killer cells and activated T lymphocytes, suggesting real induction of antitumor responses, rather than lymphocytic depletion by treatment alone. Subsequent analysis showed that a low B-cell count in blood and pre-existing antiacetylcholine receptors predicted the development of myositis in these patients; interestingly, patients who developed myositis had no specific antibodies before and after avelumab treatment [57].

### Single case reports

Outside of clinical trials, single case observations have been reported: a patient with type B2 thymoma treated with pembrolizumab who experienced severe mucocutaneous toxicity involving skin, mouth, esophagus, uvea and glands [48]; a patient with type B2 thymoma treated with a single dose of nivolumab who developed fatal myositis and myocarditis [58]; four patients who received nivolumab as second- or third-line treatment for PD-L1-positive thymic carcinoma with prolonged response or disease control [50]; and one patient with squamous cell thymic carcinoma who responded to pembrolizumab [49].

### Key points for clinical practice

• Immune checkpoint inhibitors may represent a new option of advanced thymic epithelial tumors, but implementation in the clinic is challenging based on the biology of those malignancies.
• Efficacy is actually in line with response and progression free survival reported with other available options in advanced disease [10].
• Toxicity is a major concern, despite systematic baseline work-up for autoimmunity, with frequent occurrence – higher than in other solid tumors – of severe autoimmune adverse events, mainly myocarditis, myositis and hepatitis, possibly favored by previous treatment with anthracyclines and radiation therapy.
• Immunotherapy is contra-indicated in type B1/B2 thymoma, is not a standard-of-care in type B3 thymoma or thymic carcinoma, and should not be delivered in an off-label setting without full disclosure of risks. Clinical trials are ongoing; in Europe, the European Organization for Research and Treatment of Cancer and the European Thoracic Oncology Platform have commenced a single-arm, multicentre, phase II study (the NIVOTHYM trial) to assess the efficacy of nivolumab alone or in combination with ipilimumab in patients with advanced, refractory type B3 thymoma or thymic carcinoma. A strict autoimmune work-up is planned (NCT03134118). A phase I/II trial with pembrolizumab in thymic carcinomas and thymomas is also underway at MD Anderson Cancer Center (NCT03295227).

It remains to be determined whether immunotherapy would provide a better efficacy/safety ratio with selection of patients using biomarkers. PD-L1 expression (negative or positive) may be a predictor, while the tumor mutation burden does not seem to be relevant. Greater completion of autoimmunity checks may be useful, as suggested by post-hoc analyses of clinical trials.

In clinical practice, it is important to ensure that the primary thymic nature of a squamous cell carcinoma is identified accurately in patients who may be misdiagnosed with primary lung squamous cell carcinoma. These patients are eligible for immune checkpoint inhibitors in a routine practice setting.

To conclude, immunotherapy creates new challenges in the field of thymic epithelial tumors. There is a need for multidisciplinary clinical management and research to improve understanding of biological and immune processes regulating the interactions between thymic epithelial cells and T cells in the non-neoplastic and tumoral thymus to prevent autoimmune adverse events of immune checkpoint inhibitors, and to identify biomarkers predicting efficacy and long-term survival.

## Funding

None declared.

## Disclosure

Dr Girard reports grants, personal fees and non-financial support from 10.13039/100002491Bristol-Myers Squibb; personal fees and non-financial support from 10.13039/100009947Merck Sharp and Dohme; personal fees and non-financial support from 10.13039/100004336Novartis; and grants and personal fees from 10.13039/100004319Pfizer.
